# Editorial: Biomaterials for Skin Wound Repair: Tissue Engineering, Guided Regeneration, and Wound Scarring Prevention

**DOI:** 10.3389/fbioe.2021.722327

**Published:** 2021-07-19

**Authors:** Ubaldo Armato, Giuliano Freddi

**Affiliations:** ^1^ Department of Burns and Plastic Surgery, 2nd People’s Hospital, Shenzhen University, Shenzhen, China; ^2^ Department of Surgery, Dentistry, Paediatrics and Gynaecology, Medical School, University of Verona, Verona, Italy; ^3^ Silk Biomaterials S.r.l., Lomazzo, Italy

**Keywords:** skin wounds, burns, ulcers, tissue engineering, biomaterial-guided regeneration, scar hypertrophy, scar retraction

Although laypersons may not realize it, the skin or integument is the largest and heaviest organ of our body. It is of the utmost importance that as a boundary organ it keeps its integrity and functions. True, the skin’s esthetic role crucially mediates social and emotional interactions. But there is much more than this: wounds due to any factor or agent impair the integument’s barrier function allowing the entry of infectious agents and the concurrent loss of blood and interstitial fluid components. Wounds’ severity is proportional to their extent and depth. Still other etiologic factors drive acute and chronic diseases also altering the skin’s barrier. Most importantly, bidirectional pathophysiological links exist between the skin and internal organs. A diseased organ can alter skin’s structure and function. For example, immune reactions to foreign or endogenous antigens or malignancy may drive a diffuse urticaria (hives). As an example of the reverse, extensive deep burn wounds both favor infections and release toxins inducing multiple organs failure and jeopardizing patients’ survival.

Prevalence of severe skin wounds varies according to countries and ongoing socioeconomic (e.g., urbanization) and political factors (skin burns—and not bullet lesions—are the commonest injuries affecting wounded soldiers). Such wounds impact on the patients’ survival odds, length of hospital stays, healing with hypertrophied scars (keloids) and functional impairments from scar retraction. Serious burns also alter patients’ body image, which results in psychological reverberations due to more difficult social and emotional interactions with third persons, and job-, and daily tasks coping-related hurdles. Moreover, chronic skin wounds or ulcers often concur with lifestyle-related metabolic disorders and are a growing worldwide issue causing a huge financial burden on healthcare systems.

Sen (2021) recently reviewed the available data on the economic impact of chronic wounds in the United States, pointing out that wound management is still an open clinical problem and that it will continue to be a substantial clinical, social, and economic challenge for the future. The coronavirus pandemic made this problem even worse, due to the dramatic disruption that caused in the healthcare systems worldwide. In fact, higher risks of more severe symptoms or even of death have been observed for patients with chronic wounds and related comorbidities left untreated or improperly managed.

The paper of Sen C.K. systematically reviews the most recent epidemiological data about the incidence of wound diseases, not only chronic wounds, but also pressure, foot, and venous ulcers, acute wounds, as well as diabetes-, obesity-, and infection-associated diseases. Moreover, it presents estimates about the percentage of population involved, healthcare costs, market size, and trend of care products consumption. As an example, here they report some data on the impact of chronic wounds, which are mostly seen in the elderly population (over 65 years). In the United States, 3% of these people have open wounds. It is foreseen that chronic wound will continue to be an increasingly persistent problem owing to the estimate of elderly population increase over the next decades. Accordingly, the wound care market is projected to reach $18.7 billion by 2027, with a Compound Annual Growth Rate (CAGR) of 6.6%.

As the author keenly suggests, several social, economic, environmental, psychological determinants are known to interact with the biology of the individual and to determine health outcomes such as disease development, wound healing, and life expectancy. Thus, for an integrated approach to be successful, investments are needed in welfare, education, healthcare, and research, with a patient-centered view aimed at establishing an interdisciplinary environment able to address the still open challenges.

Last decades’ scientific progress has made a remarkable impression on basic research attempts to clarify the intricate pathophysiological mechanisms involved in skin wound healing and on the clinical management of acute and chronic skin wounds. Both types of endeavors have been aiming at improving the clinical upshots of severe skin wounds and at mitigating their healthcare, economic, and social consequences. In keeping with these trends, this Research Topic updates the results of recent basic and applied research in the complex field of wound repair.

A first group papers explores innovative approaches to prevent or treat or mitigate wound’s hypertrophic and retracting scars and their unpleasant consequences.

To start with, the study of Liu et al. deals with the cutaneous distribution of the branches of the second dorsal metacarpal artery, which supply a skin flap frequently used to surgically repair hand and fingers wounds. Defining the courses of such cutaneous branches is crucial to the flap’s therapeutic success. The authors examined specimens from 16 subjects to assess and measure the origin, and course of the said artery branches and their relative location in the hand with respect to the dorsal branch of the radial nerve. They conclude that three clusters in the distal branches of the second dorsal metacarpal artery must be used as the flap’s pedicles to successfully repair wounds of the hand and fingers.

A second paper by Liu et al. concerns the novel preoperative use of 3D printed models to plan and conduct the surgical removal of complex hypertrophic scars, which cause multiple joint contractures and deformities. By availing themselves of cross-sectional computed tomography (CT) scan data the authors produced 3D printed reduced-scale models accurately reproducing the patients’ keloids. Preoperative simulations using such 3D printed models allowed to successfully remove each scar. Therefore, using such preoperative 3D printed models not only will allow surgeons to fully excise keloids but also will advance young doctors’ training and patients’ confidence.

Moreover, Zhang et al. tackle another clinically relevant facet of cutaneous scars. They investigated whether and how much the life quality of patients bearing facial burn scars depends on acceptance of their disability. The results showed that the overall quality of life of such patients is positively correlated with the degree of their disability acceptance. Therefore, the authors suggest that a primary therapeutic target of the medical and nursing staff is heightening the patients’ disability acceptance.

The second group of papers deals with the selection, production, and testing of novel biomaterials targeted for more therapeutically advantageous wound bandages and with the development of novel 3D biomaterial scaffolds for tissue and vascular engineering/regeneration.

Wound healing is a dynamic process going through succeeding stages deeply influenced by concurring local (e.g., infection), general (e.g., diabetes), and therapeutic (e.g., specific bandages) factors. In this framework, Wang et al. deal with the all-important topic of chronic wounds due to diabetes, infections, or radiation exposure, which altogether constitute a worldwide growing medical problem. The authors mention recent studies showing that physical cues significantly impact on both stem cells fate and bacterial resistance. Therefore, it would be beneficial to tune up the physical properties of the biomaterials—particularly of those made of nanoparticles (NPs) —used for chronic wounds. To improve the latters’ care and healing upshots, the authors analyze the tunable physical (i.e., mechanical-, structural- and size-related) properties of both dermal matrixes and wound bandages. Moreover, they discuss the physical and structural factors governing stem cells’ fate. Next, they survey wound dressings according to the size-related properties of their components (i.e., metal NPs, lipid NPs, and polymeric NPs). Finally, they focus on the known problem caused by AgNPs cytotoxicity proposing size-, dose-, and time-dependent solutions to prevent it.

From another relevant standpoint, Shi et al. examine the rational criteria that should govern an optimal selection of dressings for each type of wounds in clinical settings. To improve the readers’ understanding of the topic, they summarize wound dressings’ developmental history and classify the current bandage’s types. Next, they expound the clinical guidelines to select the best wound dressings according to wound types**.** In doing this, they focus on burn wounds and on chronic ulcers caused by diabetes, pressure, and/or varicose veins. Usefully, the authors discuss in detail the pros and cons underlying the dressing choices for each wound type.

Two more contributions examine the clinical effectiveness and safety of hydrogel-based dressings as skin wounds therapeutics. On the one hand, Zhang et al. undertake a systematic review and meta-analysis of 43 clinical trials that compared hydrogel bandages *vs.* non-hydrogel dressings. The results showed that the hydrogel dressings were therapeutically advantageous under several respects, including pain alleviation, both for acute and chronic skin injuries. Therefore, the authors posit that hydrogel dressings are effective and safe therapeutics for skin wounds management. On the other hand, Wang et al. describe a novel set of composite hydrogels made of oxidized and/or hydrazide hyaluronic acid (HA) and quaternized chitosan (QCS). The authors investigated whether HA/QCS hydrogels did prevent bacterial infections and promote the healing of seawater-immersed wounds. The results of *in vitro* and *in vivo* tests showed that HA/QCS hydrogels were biocompatible and inhibited bacterial growth. *In vivo,* these hydrogels also efficiently promoted the repair of seawater-immersed wounds. Therefore the authors postulate that HA/QCS hydrogels advance the healing of chronic wounds from various causes.

Concerning another field of endeavor, Mulholland provides recent details about the electrospinning technique that rapidly and cost-effectively allows to produce nanofibers from a wide variety of (bio)polymers. The nanofibers’ exceedingly high surface area makes them excellent components of novel wound dressings. The author points out that using electrospun biomaterials can confer an enhanced tensile strength to the engineered/regenerated skin and prevent or mitigate hypertrophic scar development. Since electrospun biomaterials hold such enticing promises, the author suggests refining the electrospinning technique to combine it with innovative gene therapies to boost tissue repair and minimize scarring.

In the same context, Marsi et al. report on the electrospinning of a classical biomaterial component, i.e., poly(lactic acid) (PLA) combined with titanium dioxide (TiO_2_). PLA is biodegradable, biocompatible, easy to manage, and suitable to form composites. Due to its favorable characteristics and low cost, the use of PLA for skin tissue engineering/regeneration has been increasing with time. The authors prepared electrospun PLA/TiO_2_ nanofibrous composites and minutely analyzed their properties and biocompatibility using various *in vitro* and *in vivo* preclinical assays. Importantly, the PLA/TiO_2_ nanopolymers exhibited a nanoporous structure mimicking cellular environments and were not cytotoxic at all. Altogether, the results supported the view that PLA/TiO2 nanomembranes may hinder scarring.

Purified silk fibroin (SF), a biomacromolecule produced by domesticated *Bombyx mori* silkworm and other insects, is also amenable to electrospinning. SF lends itself to be formed as 2D membranes or 3D scaffolds made of microfibers or nanofibers, or hydrogels. SF’s popularity as biomaterial is due to a remarkable set of features. First, SF is highly biocompatible and rightly so: about 50 human proteins own conserved homology sequences of *Bombyx mori*’s SF (Armato et al., 2011). Second, SF is neither persistently inflammagenic nor profibrogenic nor cytotoxic. Third, once grafted SF does not induce any foreign body response (Dal Prà et al., 2005; Chiarini et al., 2016). Fourth, SF-adhering human fibroblasts cultured *in vitro* persistently release exosomes carrying heightened amounts of various angiogenic factors (Hu et al., 2021). Obviously, to become manifest all these advantageous properties strictly require using a very highly purified SF. In this context, Alessandrino et al. report on the properties of a newly multi-layered SF tubular scaffold, named SilkGraf, designed to substitute, repair, or regenerate small peripheral arteries. SilkGrafs samples kept for 20 days *in vitro* promoted the adhesion, survival, metabolism, and growth of human coronary artery endothelial cells, aortic smooth muscle cells, and aortic adventitial fibroblasts. Notably, the SilkGraf-attached aortic fibroblasts synthesized much smaller amounts of type I procollagen than they did on polystyrene, further strengthening the view (Dal Prà et al., 2005; Chiarini et al., 2016) that SF hinders fibrogenesis and hypertrophic scarring. Moreover, the SilkGraf-attached three cell types did not secrete any proinflammatory cytokines. In addition, SilkGraf samples exhibited a good hemocompatibility. Finally, *in vivo* pilot trials identified the sheep as the animal model apt for future medium-to-long term preclinical trials with SilkGraf.

## Conclusion

On a personal note, I wish to state that editing these paper in this Research Topic was profitable under both the scientific and human relationship standpoints. On a more general note, I expect that this Research Topic of innovative research works will inspire the readers to conceive, produce, and experimentally evaluate novel dermal matrixes and/or wound dressings based upon even more advanced design strategies to further improve skin wounds’ therapeutic management.
